# The impact of dornase alfa on imaging features of bronchiectasis

**DOI:** 10.21203/rs.3.rs-6991583/v1

**Published:** 2025-09-03

**Authors:** Christina M. Mingora, Federico Mollica, Daan Caudri, Punitkumar Makani, Harm Tiddens, Caroline Brailsford, Patrick A. Flume

**Affiliations:** Medical University of South Carolina; Erasmus MC – Sophia Children’s Hospital; Erasmus MC – Sophia Children’s Hospital; Erasmus MC – Sophia Children’s Hospital; Thirona B.V; Medical University of South Carolina; Medical University of South Carolina

**Keywords:** bronchiectasis, dornase alfa, image analysis

## Abstract

**Background:**

Nebulized dornase alfa is an approved therapy for persons with cystic fibrosis, but not for other causes of bronchiectasis. Nonetheless, dornase works by breaking down extracellular DNA in airway phlegm and should be effective in people with bronchiectasis who have excessive purulent phlegm. The purpose of this study was to look at radiographic changes in persons with bronchiectasis (not CF) treated with dornase alfa.

**Methods:**

This is a single-center retrospective review of clinical data and analysis of images after initial treatment with dornase alfa. Eligible subjects included those with documented bronchiectasis who were treated with dornase alfa as part of their clinical care and who had chest computed tomography performed prior to and shortly after initial treatment. Descriptive clinical data are provided but not analyzed. Images were analyzed using validated methods including an artificial-intelligence-based algorithm to assess measures of bronchiectasis, airway wall thickness, and mucus plugs comparing before and after treatment.

**Results:**

Eleven subjects were treated with dornase alfa without complication and with improved symptoms and reduced numbers of pulmonary exacerbations. One subject had improvement in pulmonary function. There was a nominal reduction in a measure of bronchiectasis and the number and volume of mucus plugs after dornase alfa compared to pre-treatment.

**Conclusions:**

This novel image analysis of the impact of dornase alfa on features of bronchiectasis is consistent with the improved clinical symptoms reported by the subjects. These observations may help define better criteria to predict which bronchiectasis subjects are most likely to benefit from dornase alfa therapy.

## Introduction

Bronchiectasis (BE) is a pulmonary condition manifested by irreversible dilated airways (1). Although there are many etiologies that can cause or associate with BE, a common feature of the condition is impaired mucociliary clearance (1). For many patients this permits persistent infection by opportunistic pathogens and an exaggerated inflammatory response (2) resulting in chronic symptoms of cough and sputum production, and episodic worsening of symptoms called pulmonary exacerbations (3). To date, there are no medications with the labeled indication for BE except for those approved for cystic fibrosis (CF), which include inhaled antibiotics and dornase alfa (4). These therapies have been tested in trials including subjects with BE due to causes other than CF, but none have met success sufficient to result in a change to the approved indications (5, 6).

Criticism of these trials have suggested that they did not enroll subjects with the clinical phenotype expected to benefit from the therapies (7). Yet there are reports that have described patients with BE who have benefited from their use (7, 8). Treatment guidelines have even included a recommendation for inhaled antibiotics based on these clinical reports (9). However, treatment guidelines have not recommended dornase alfa based on the results of a clinical trial in BE patients that did not demonstrate efficacy but also reported there were adverse outcomes when compared to placebo (5). This was a surprising finding since adverse events due to dornase in persons with CF are rarely reported, including use in infants and children.

For dornase alfa to be effective in BE, there must be substrate for which the drug can work (i.e. excessive amounts of DNA present within the airways phlegm). This could be predicted by the presence of mucus plugs and increased airway wall thickness on computed tomography of the chest (chest CT) (10). We report our experience with inhaled dornase alfa in patients with BE who were having increased symptoms despite other therapies, with particular emphasis on the radiologic findings on chest CT performed before and after use of dornase alfa.

## Methods

This is a retrospective review of clinical data and analysis of images performed at a single center. Eligible subjects included those with documented BE by chest CT who had been treated with dornase alfa as part of their clinical care (i.e. not a research protocol) for at least one month. For the chest CT analysis, they must have chest CT images performed prior to and shortly after treatment with dornase alfa. Subjects with CF were excluded.

The variables of interest included demographic data, pulmonary function testing, symptoms related to bronchiectasis, history of pulmonary exacerbation, culture results from respiratory specimens, and concomitant medications (specifically for airways disease). Dates of testing and start of dornase alfa were documented. Measures collected most proximally to before treatment and after at least one month of treatment were used for comparisons.

### BEST-CT Analysis

CT scans were analyzed using the validated Bronchiectasis Scoring Technique for CT (BEST-CT) (10). The subscores of BEST-CT are expressed as percentages of total lung volume. The items scored are atelectasis/consolidation, BE with and without mucus plugs (MP), airway wall thickening (AWT), ground-glass opacities (GGO), bullae, airways, and parenchyma. Composite scores were calculated: total BE (i.e., BE with and without MP), and total disease (i.e., all items but airways and parenchyma).

### LungQ Analysis

Chest CT images were analyzed using validated artificial intelligence-based algorithms (LungQ^®^, Thirona, Nijmegen, The Netherlands) (11, 12). A fully automatic Mucus Plug (MP) and Bronchus-Artery (BA) analysis was used to quantify the number and volume of MP and BA dimensions and ratios. LungQ segments the bronchial tree and identifies for each bronchus the matching artery. For each BA-pair it computes the following diameters: bronchus outer and inner diameter (B_out_ and B_in_), arterial diameter(A), and bronchus wall thickness (B_wt_). It then computes the following ratios: B_out_/A, B_in_/A, B_wt_/A, and B_wa_/B_oa_ (bronchus wall area/bronchus outer area) for each segmental generation. We compared volume and number of MP and the median of all BA-dimensions and ratios B_out_/A, B_in_/A, B_wt_/A, and B_wa_/B_oa_ for segmental generations 1 to 6 (G_1–6_). We defined a B_out_/A > 1.1 (= 95th percentile of normal subjects) to be indicative of bronchiectasis (12); we also looked at a more conservative measure of B_out_/A > 1.5(13). Increased airway wall thickness was defined as a B_wt_/A > 0.14. As an additional measure, one that is independent of the A dimension, we defined B_wa_/B_oa_ 0.34 as the upper limits of normal. Finally, we recorded the number and volume of mucus plugs. We performed non-parametric Wilcoxon test (paired t-test) for comparisons.

## Results

### Subjects

Eleven subjects had been treated with dornase alfa and are included in the clinical analysis. All subjects were women, and their demographic data are shown in Table 1. All subjects remained on treatment ranging from one to nearly nine years; two of these subjects had stopped treatment briefly because of insurance issues. One subject died 1.5 years following start of treatment.

### Clinical outcomes

All subjects reported at least one persistent respiratory symptom prior to starting dornase (Table 1). Six subjects (55%) were hospitalized at least once (total 9 hospitalizations) for treatment of a pulmonary exacerbation in the year prior to treatment with dornase alfa. Review of notes in the first clinic visit after initiation of dornase alfa described clinical improvement in all subjects except one for whom there was no reported change in symptoms. In the year following the initiation of dornase alfa, four subjects had been hospitalized (total 5 hospitalizations) for treatment of a pulmonary exacerbation. Three subjects had pulmonary function testing both before and after treatment; two experienced no change and one experienced a moderate improvement in FEV_1_ (47 to 58 percent predicted) but not FVC. There were no adverse events attributed to dornase alfa.

### Radiographic measures

Baseline CT chest was performed a median of one month (range 0–7 months) prior to starting dornase alfa in all subjects; one subject was excluded because of poor image quality. Seven subjects had subsequent images obtained shortly after starting treatment (median 1 month; range 1–2 months) and are included in the comparative analysis.

**Figure 1:**
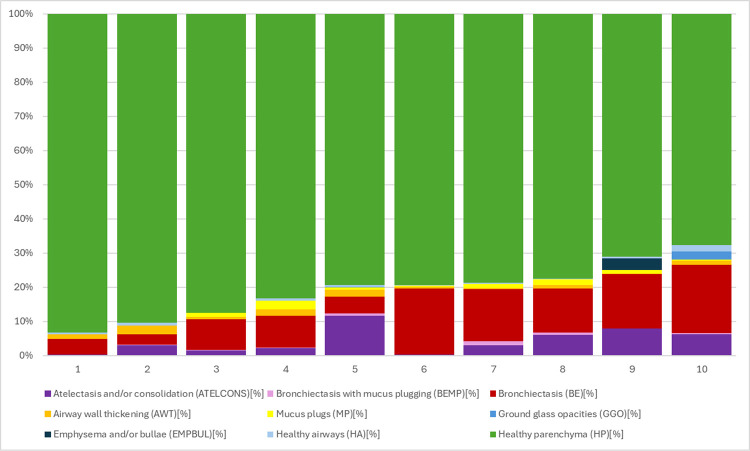
BEST-CT Total DIS% pre-dornase (7 CTs) BEST-CT manual analysis of CT images of all subjects prior to starting dornase alfa. There is heterogeneity among them, but each had a large amount of lung affected by bronchiectasis and detectable increased airway wall thickness and mucus plugs.

The scores from the BEST-CT manual analysis of the baseline images for all subjects (n = 10) are shown in Fig. 1; all subjects had a large proportion of lung affected by bronchiectasis as well as detectable increased airway wall thickness and mucus plugging. The changes in BEST scores for those subjects who had follow up imaging (n = 7) are shown in Table 2. There were nominal improvements in total bronchiectasis, bronchiectasis with mucus plugs, airway wall thickness, mucus plugs, and healthy parenchyma, although none were statistically significant.

**Figure 2:**
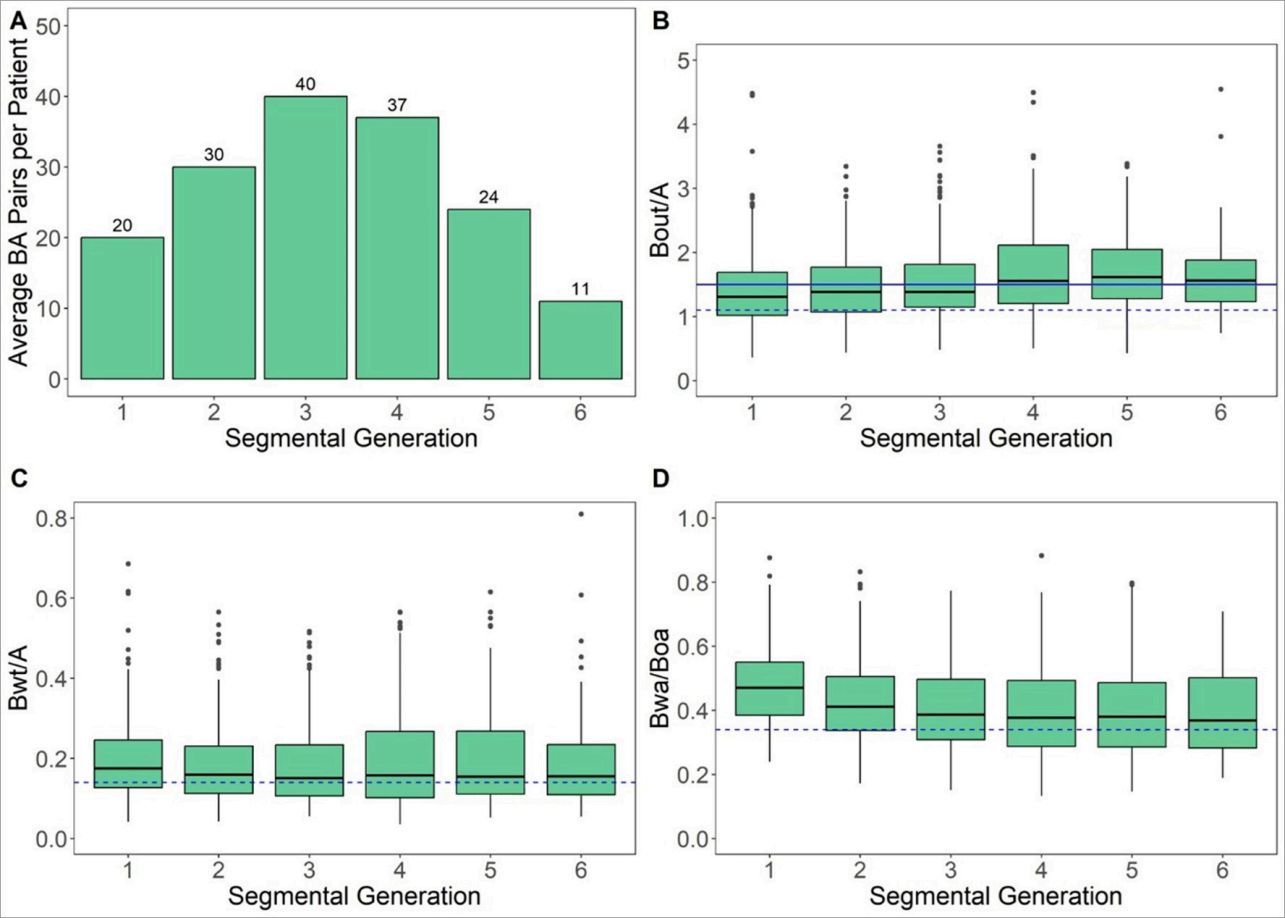
(**a**) average number of airway-artery pairs per segmental generation 1 to 6 (1 = subsegmental bronchus). (**b**) distributions of airways based on the outer diameter of the bronchus (B_out_) and the adjacent artery(A), or B_out_/A; threshold ratios of 1.1 (=95^th^ percentile of normal subjects) (12), shown in dashed line, or a more conservative threshold of 1.5(13), shown in solid line, are considered indicative of bronchiectasis. (**c**) distributions of bronchus wall thickening (B_wt_) relative to the adjacent artery (A); a B_wt_/A ratio above0.14 (=95^th^ percentile of normal subjects), shown in dashed line, can be considered indicative of increased bronchial wall thickening [Lv 2023]. (**d**) distributions of bronchus wall area (B_wa_) relative to the bronchus outer area (B_oa_); a B_wa_/B_oa_ ratio above 0.34 (=95^th^ percentile of normal subjects), shown in dashed line, can be considered indicative of increased bronchial wall thickening [Lv 2023].

The LungQ-BA detected a mean of ~ 142 BA pairs from G_1 – 6_ in the baseline images (n = 10). The average number of BA pairs per patient and per segmental generation are shown in Fig. 2. The extent of bronchiectasis is demonstrated graphically in Fig. 2; the proportion of airways meeting criteria for bronchiectasis is high (increased B_out_/A > 1.1 ~ 62%) and 44.9% with the more conservative estimate of B_out_/A > 1.5. There was also increased airway wall thickness using both measures (B_wt_/A > 0.14 and B_wa_/B_oa_ >0.34). The complete descriptive statistics for the baseline images are shown in Table 3.

For the comparative analysis (n = 7) we were able to detect a nominal reduction in the measure of bronchiectasis (B_out_/A) following dornase alfa, including the proportion of airways above the thresholds we used to define bronchiectasis, but they did not achieve statistical significance (Table 3). There was also a nominal increased in the overall volume of the lung after dornase alfa compared to prior (Table 3) but this was also not significant. For assessment of increased airway wall thickness, there was no improvement with one measure (B_wt_/A) but a nominal reduction in the other (B_wa_/B_oa_). There were also reductions in the average number of mucus plugs (~ 20%) and their volume (~ 40%), but without achieving statistical significance. The subject who experienced improvement in pulmonary function testing also had the greatest impact on the number (108 pre and 39 post) and volume (9.2 ml pre and 2.6 ml post) of mucus plugs.

## Discussion

We have reported our results on a small cohort of subjects with bronchiectasis (not CF) whose symptoms responded favorably to inhaled dornase alfa. The decision to use dornase alfa in these subjects was based on the clinical determination that they were not thriving in response to standard therapy, and we wanted to evaluate their response (e.g. n = 1 experiment) to a drug proven effective in patients with bronchiectasis due to CF. We monitored the patients carefully assessing their symptoms as well as changes in CT imaging. We admit that this is a highly biased cohort in that these subjects were selected as candidates for treatment for clinical reasons and were able to tolerate the dornase alfa for at least one month, yet the subjects remained on this therapy for an extended period and there was a reduction in the number of pulmonary exacerbations in the year following start of treatment.

In addition to our subjects reporting symptomatic improvement, there were nominal objective improvements following one month of treatment as measured on imaging by two methods assessing bronchiectasis, airway wall thickness, and mucus plugs; we did not expect to find statistical significance given the small sample size. The nominal improvement in measures of bronchiectasis (B_out_/A) is unexpected as structural changes should not change with such short-term therapy; however, this could be related to the nominal increase in lung volumes between the scans (14). What should be expected if dornase alfa is to be successful is a reduction in the number and volume of mucus plugs and perhaps a reduction in airway wall thickness as this may represent an decrease in airway phlegm. There was a reduction in the mucus plugs (number and volume), and in one subject the change was considerable and associated with an improvement in pulmonary function. The impact on airway wall thickness was less remarkable as there was no change in one measure (B_wt_/A) but an increase in another (B_wa_/B_oa_), the latter perhaps of greater interest as it is independent of the airway diameter. It is not yet known what quantity of change for any of these parameters would be considered clinically significant, but other studies have shown a reduction in airway wall thickness of 3% in response to hypertonic saline in young children (15), a 3% reduction in response to azithromycin in infants with CF (16), and 12% reduction in adults in response to CFTR modulators (17).

With respect to why previous trials of therapies for bronchiectasis have failed, a concern has been raised that the subjects enrolled in those trials may not have been best suited for the treatment. Enriching trials with subjects carefully selected to be the most likely to respond requires inclusion criteria that are most predictive of a response. Our indication for clinical use of dornase alfa in these subjects was persistent symptoms and/or recurring pulmonary exacerbations but who also had CT chest findings of increased airway wall thickness and mucus plugs. This was a subjective approach to assessment of subjects but is justified based on this retrospective assessment of our subjects’ imaging using a novel automated objective image analysis.

These observations suggest there are patients with bronchiectasis (CF or not) who may benefit from inhaled dornase alfa, but we need better criteria to define who is most likely to benefit. This is similar to studies of inhaled antibiotics in bronchiectasis subjects with the intent to reduce the rate of pulmonary exacerbations that failed because the exacerbation rate in the placebo group was much lower than predicted (18). A careful examination of the demographic data of these failed trials demonstrates a low use of macrolides, shown to reduce pulmonary exacerbations in bronchiectasis (9), and a high proportion of subjects reported to have asthma and/or COPD raising the question as to whether they were appropriate candidates for the inhaled antibiotic. The study of dornase alfa enrolled a large number of subjects but very few demographic data were reported (5); no medications were included in the publication but there was a high rate of tobacco use. Sputum DNA is reported but the methods of analysis are not, and the values are means with a broad range provided. Our conclusion is that there is insufficient information upon which to base the outcomes, but our suspicion is that this trial, like others that followed, enrolled the wrong phenotype.

We are not proposing broad use of dornase alfa for the bronchiectasis population but suggest that carefully selected patients may indeed benefit from this therapy. Novel methods to identify subjects, such as objectively evaluating CT chest images, may play an important role in defining eligible subjects as well as demonstrating benefit following therapy.

## Supplementary Material

Supplementary Files

This is a list of supplementary files associated with this preprint. Click to download.

- Table1.docx

## Figures and Tables

**Table 2:** BEST-CT outcomes

Feature as percentage of total lung volume	Pre-dornase, mean (SD)	Post-dornase, mean (SD)
Total disease	22.7 (5.0)	20.4 (13.0)
Total bronchiectasis	14.4 (5.3)	10.9 (6.1)
Atelectasis and/or consolidation	5.3 (3.8)	7.1 (8.5)
Bronchiectasis with mucus plugging	0.5 (0.5)	0.2 (0.2)
Bronchiectasis	14.0 (5.4)	10.7 (6.0)
Airway wall thickness	1.0 (0.7)	0.8 (0.6)
Mucus plugging	1.1 (0.8)	0.8 (1.0)
Ground glass opacity	0.4 (0.8)	0.4 (1.0)
Healthy airways	0.6 (0.6)	0.2 (0.2)
Healthy parenchyma	76.7 (5.4)	79.3 (13.1)

**Table 3:** LungQ BA-analysis descriptive statistics BA-pairs

	All subjects (n = 10)	Subjects with pre and post CT images (n = 7)	
Measure, mean (SD)		Pre-dornase	Post-dornase
Inspiratory lung volume (ml)	3996.7 ± 1367.5	4121.6 ± 1089.5	3992.1 ± 1015.6
BA pairs G_1 − 6_	141.6 ± 90.7	160.0 ± 89.9	131.0 ± 66.2
B_out_/A	1.29 ± 0.4	1.39 ± 0.4	1.30 ± 0.3
B_in_/A	0.96 ± 0.4	1.06 ± 0.4	0.96 ± 0.3
B_out_/A %>1.1	62.1 ± 33.8	70.1 ± 32.4	62.6 ± 18.8
B_out_/A %>1.5	37.2 ± 23.5	44.9 ± 22.6	39.3 ± 17.6
B_wt_/A	0.16 ± 0.05	0.16 ± 0.05	0.16 ± 0.06
Bwa/Boa	0.45 ± 0.13	0.42 ± 0.09	0.45 ± 0.14
B_wt_/A % of pairs > 0.14	54.5 ± 27.8	54.0 ± 23.2	57.6 ± 27.7
B_wa_/B_oa_ % of pairs > 0.34	75.2 ± 27.0	72.9 ± 27.9	63.9 ± 37.8
Mucus plugs, number	22.8 ± 31.8	28.3 ± 36.6	22.3 ± 23.5
Mucus plugs, volume (ml)	1.61 ± 2.76	2.08 ± 3.22	1.23 ± 1.36

## Data Availability

The datasets used and/or analysed during the current study are available from the corresponding author on reasonable request.

## Abbreviations

A: arterial diameter
AWT: airway wall thickening
B_in_: bronchus inner diameter
B_oa_: bronchus outer area
B_out_: bronchus outer diameter
B_wa_: bronchus wall area
B_wt_: bronchus wall thickness
BA: bronchus-artery
BE: bronchiectasis
CF: cystic fibrosis
COPD: chronic obstructive pulmonary disease
CT: computed tomography
DNA: deoxyribonucleic acid
BEST: Bronchiectasis Scoring Technique
FEV_1_: forced expiratory volume at 1 second
FVC: forced vital capacity
G_1–6_: segmental generations 1 to 6
GGO: ground-glass opacities
MP: mucus plugs
